# Visual Sensor Technology for Advanced Surveillance Systems: Historical View, Technological Aspects and Research Activities in Italy

**DOI:** 10.3390/s90402252

**Published:** 2009-03-30

**Authors:** Gian Luca Foresti, Christian Micheloni, Claudio Piciarelli, Lauro Snidaro

**Affiliations:** Department of Mathematics and Computer Science University of Udine, via delle Scienze, 206, 33100 Udine, Italy

**Keywords:** Advanced visual surveillance, visual sensor networks, active vision, object detection, tracking, human behaviour understanding

## Abstract

The paper is a survey of the main technological aspects of advanced visual-based surveillance systems. A brief historical view of such systems from the origins to nowadays is given together with a short description of the main research projects in Italy on surveillance applications in the last twenty years. The paper then describes the main characteristics of an advanced visual sensor network that (a) directly processes locally acquired digital data, (b) automatically modifies intrinsic (focus, iris) and extrinsic (pan, tilt, zoom) parameters to increase the quality of acquired data and (c) automatically selects the best subset of sensors in order to monitor a given moving object in the observed environment.

## 1. Introduction

In the last years, there has been a growing interest in surveillance applications due to the increasing availability of cheap visual sensors (e.g., optical and infrared cameras) and processors. In addition, after the events of September 11th, 2001, citizens are demanding much more safety and security in urban environments. These facts, in conjunction with the increasing maturity of algorithms and techniques, are making possible the use of surveillance systems in various application domains such as security, transportation and automotive industry.

The surveillance of remote and often unattended environments (e.g., metro lines and railway platforms, highways, airport waiting rooms or taxiways, nuclear plants, public areas, etc.) is a complex problem implying the cooperative use of multiple sensors. Surveillance systems have provided several degrees of assistance to operators and evolved in an incremental way according to the progress in technology and sensors. Several kinds of sensors are nowadays available for advanced surveillance systems: they range from tactile or pressure sensors (e.g., border surveillance) to chemical sensors (e.g., industrial plant surveillance or counter terrorism activities) to audio and visual sensors.

For monitoring wide outdoor areas, the most informative and versatile sensors are the visual ones. This information can be used to classify different kinds of objects (e.g., pedestrians, groups of people, motorcycles, cars, vans, lorries, buses, etc.) moving in the observed scene, to understand their behaviours and to detect anomalous events. Useful information (e.g., classification of the suspicious event, information about the class of detected objects in the scene, b/w or colour blob of the detected objects, etc.) can be transmitted to a remote operator for augmenting its monitoring capabilities and, if necessary, to take appropriate decisions.

The main objective of this paper is to analyze the technological aspects of advanced visual-based surveillance systems, with particular emphasis on advanced visual sensor networks that (a) directly process locally acquired digital data, (b) automatically modify intrinsic (focus, iris, etc.) and extrinsic (pan, tilt, zoom, etc.) parameters to increase the quality of acquired data and (c) automatically select the best subset of sensors in order to monitor (detect, track and recognize) a given moving object in the observed environment.

A major requirement in automated systems is the ability to self-diagnose when the video data is not usable for analysis purposes. For instance, when video sensors cameras are used in an outdoor application it is often the case that during certain times of the day there is direct lighting of the camera lens from sunlight; a situation that renders the video useless for monitoring purposes. Another example of such a scenario is a weather condition such as heavy snowfall during which the contrast levels are such that people detection at a distance is rather difficult to do. Thus in these scenarios, it is useful to have a system diagnostic that alerts the end-user of the unavailability of the automated intelligence functions. Ideally the function that evaluates the unavailability of a given system should estimate whether the input data is such that the system performance can be guaranteed to meet given user-defined specifications. In addition, the system should gracefully degrade in performance as the complexity of data increases. This is a very open research issue that is crucial to the deployment of these systems.

The paper is organized as follows. Section 2. presents a brief historical view of visual-based surveillance systems from the origins to nowadays and a short description of the main research projects in Italy on surveillance applications in the last twenty years. Section 3. describes the main technological aspects of the last generation of intelligent visual-based surveillance systems.

## 2. Advanced Visual-based Surveillance Systems

Visual surveillance systems were born in 1960 where CCTVs became available on the market by providing data at acceptable quality. According to the classification proposed by Regazzoni et al. [1] video surveillance systems that have been proposed in the literature can be classified under a technological perspective as belonging to three successive generations.

### 2.1. Evolution of Visual-Based Surveillance Systems

**Figure 1.**
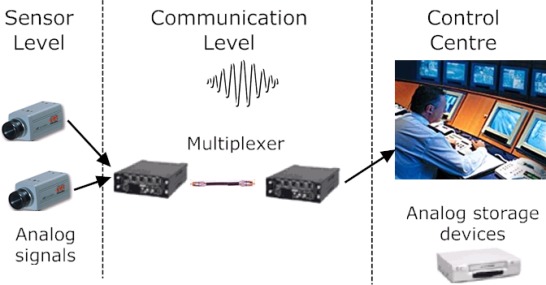
Example of the general architectural of a video-based surveillance system of the first generation (1960–1980).

First video surveillance systems (1960–80) used multiple analog video cameras (sensor level) to monitor indoor or outdoor environments by transmitting and displaying analog visual signals in a remote control room (Figure 1). Multiple video signals were presented to the human operator after analog communication (local processing level) through a large set of monitors. Video streams were normally stored on analog storage devices (i.e., VHS, etc.).

**Figure 2.**
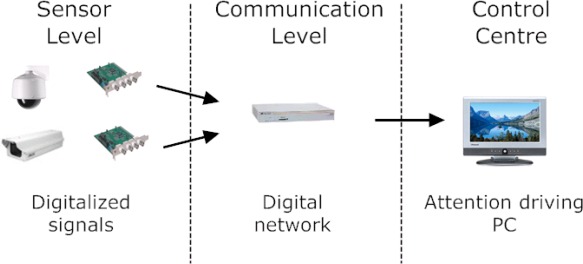
Example of the general architectural of a video-based surveillance system of the second generation (1990–2000).

These systems suffered of (a) limited attention span of the operators that may result in a significant rate of missed events of interest or alarms [2], (b) high bandwidth requirements that limits the number of sensors to be used [3] and (c) large amount of tapes to be stored that transform the off-line archival and retrieval of video frames containing events of interest in a complex operation. During the period 1980–90 the fast development of electronic systems allowed to increase the performances of video cameras, personal computers and communication technologies. In particular, advanced video cameras characterized by higher image resolution, low cost personal computers and more robust and less expensive communications links become available on the market. In this period, second generation surveillance systems (1980–2000) became a reality (Figure 2). The main characteristics of these systems were the use of digital video communications and the use of simple automatic video processing procedures able to help the operator in detecting some simple interesting events. Several important research papers have been published in that period describing results in real-time detection and tracking of moving objects in complex scenes [4], human behaviour understanding [5, 6, 7, 8, 9] intelligent man-machine interfaces [10, 11] wireless and wired broadband access networks [12], video compression and multimedia transmission for video based surveillance systems [13, 14].

**Figure 3.**
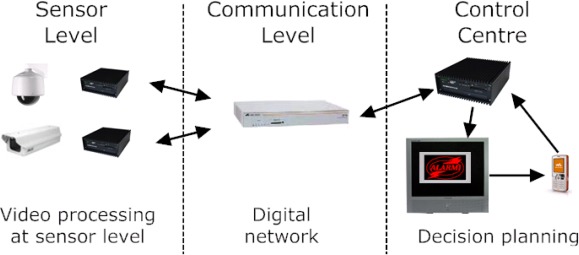
Example of the general architectural of a video-based surveillance system of the third generation.

The second generation of surveillance systems reached only partially full digital video signal transmission and processing [13, 15, 16, 17, 18, 19, 20, 21, 22, 23]. Few system subparts use digital methods to solve communication and processing problems. In the first years of the third millennium, studies began for providing a ”full digital” design of video-based surveillance systems, ranging from the sensor level up to the presentation of adequate visual information to the operators (Figure 3). In this new architecture model, advanced video cameras constitute the sensor layer while different advanced transmission devices using digital compression form the local processing layer. An intelligent hub able to integrate data coming from multiple low-level layers constitute the main component of the network layer, where all communications are in digital form. Finally, an advanced Man-Machine Interface (MMI) assists the operator by focusing his attention to a subset of interesting events and possible pre-alarms.

Research activity in the field of advanced visual-based surveillance systems is today mainly focused on three different directions: (a) to design and develop new embedded digital video sensors and advanced video processing/understanding algorithms, (b) to design and develop new sensor selection and data fusion algorithms, (c) to design and develop new high bandwidth access networks.

The design of new embedded digital video sensors is moving in the direction of directly process locally acquired digital data at sensor level [24]. Hundreds of video sensors are organized in wireless networks [25, 26] that must collect data in real-time and send to the control centre only video streams of interesting events. Due to the relatively high power consumption characteristics of cameras adequate procedures must be developed to devise energy-aware resource [27]. Recently, power-hungry camera nodes have been studied integrating a CMOS camera and a micro-controller, but the image resolution, memory and computational power are not completely adequate for the visual tasks [28]. The design and development of new sensor selection and data fusion algorithms allow to choose for a given event the best set of sensors from a large sensor network. Moreover, high bandwidth access networks allow to integrate both homogeneous and heterogeneous data coming from sensors spatially distributed over wide areas. In order to reduce the bandwidth requirements, encoding can be used to reduce the amount of visual data to be transmitted by using methods that exploit spatial-temporal information (e.g. MPEG, M-JPEG, H263, etc.) or that can be applied separately to video frames (e.g. JPEG). However, this operation requires more processing by increasing net energy consumption.

### 2.2. Visual-Based Surveillance Systems in Italy

**Figure 4.**
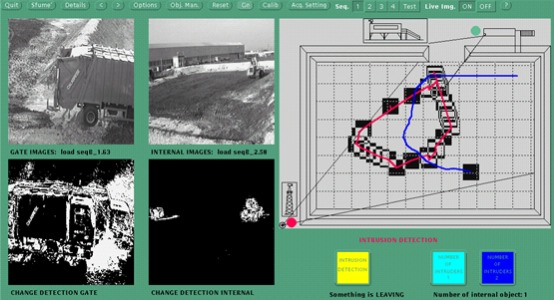
Man-machine interface of the visual-based surveillance system developed within the ATHENA project (1994–1998).

Starting from 1990 interesting second generation video-based surveillance systems have been studied in the context of different international (e.g. VSAM Program, USA) and European research programs (e.g. ESPRIT Program, European Union). Some Italian industries and Universities have participated to these research programs and have carried out prototypical demonstrators installed in real environments. The first International project on visual-based surveillance system with the participation of Italian partners (IRST and University of Genoa) was the EEC-ESPRIT II P5345 DIMUS (Data Integration in Multisensor Systems) project, in 1990–92. The aim of the project was the development of a multisensor surveillance system for the remote monitoring of metro line stations. Video cameras and acoustic sensors were integrated into a common framework able to help the operator of a remote control centre to detect some interesting events (e.g., people beyond the yellow line on the platform, crowed platform when the train is arriving, gunshots, etc.). A demonstrator was installed in the Genoa-DiNegro metro line station [29]. Another interesting international project on the subject of visual-based surveillance system with the participation of Italian partners (Technopolis Csata, Assolari Nuove Tecnologie, University of Genoa) was the CEC-ESPRIT 6068 ATHENA (Advanced Teleoperation for Earthwork Equipment) project, developed in 1994–1998. The aim of the project was the automation of earthwork operations within waste disposal sites. The objective was to operate an unmanned vehicle with autonomous capabilities, and to equip the control station with advanced teleoperation capabilities. In particular, the visual surveillance system was in charge of the following tasks: (a) intruder detection in the sanitary landfill (monitored area) by using image processing techniques applied to b/w images acquired by multiple video cameras, (b) real-time tracking of detected intruders inside the monitored area and (c) collision avoidance. Figure 4 shows a frame of the MMI of the ATHENA visual surveillance system.

An exhaustive description of the visual-based surveillance system developed in the ATHENA project can be found in [30].

Italian participation in international projects on visual surveillance can be pointed out also in the ESPRIT Project 2152 VIEWS (*Visual Inspection and Evaluation of Wide-area Scenes*), in the ESPRIT Project 8483 PASSWORDS (*Parallel and real-time Advanced Surveillance System With Operator assistance for Revealing Dangerous Situations*), in the IST-1999-11287 project ADVISOR (*Annotated Digital Video for Surveillance and Optimised Retrieval*), in the IST-1999-10808 project VISOR BASE (*VIdeo Sensor Object Request Broker open Architecture for Distributed Services*) and in the IST-2007-045547 project VIDIVideo (*Interactive semantic video search with a large thesaurus of machine-learned audio-visual concepts*).

**Figure 5.**
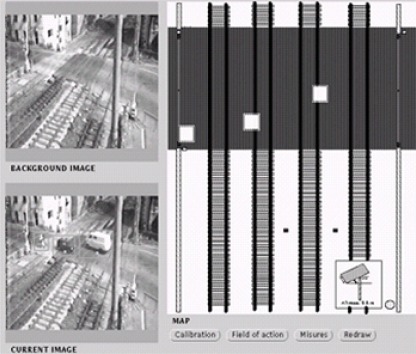
Man-machine interface of the visual-based surveillance system developed within the Italian PFT2 project (1993–1996).

The VIEWS project was a reliable application of real time monitoring and surveillance of outdoor dynamic scenes in constrained situations. Two were the driving applications of the VIEWS project: ground traffic surveillance of civil airports and traffic surveillance of public roads. The main goal of this project was the detection, tracking and classification of static or moving object in the observed scene. The goal of the PASSWORDS project was to design and develop an innovative prototype of a real-time image analysis system for visual surveillance and security applications, based on low-cost hardware and distributed software. The main functional objectives consisted in: (a) detection of motion in specific areas; (b) stopped objects detection; (c) crowd (people, cars, etc.) flow measurement and quantitative estimation; (d) crowd shape-and-structure analysis (e.g., behaviour of persons or groups, etc.). Two pilot applications have been used to demonstrate the functionalities of the system: the surveillance of an outdoor light metro network and the surveillance of a supermarket and its surroundings [31]. The ADVISOR project was addressing the environmental and economic pressures for increased use of public transport. Metro operators need to develop efficient management systems for their networks using CCTV for computer-assisted automatic incident detection, content based annotation of video recordings, behaviour pattern analysis of crowds and individuals, and ergonomic human computer interfaces. The VISOR BASE project was aimed at creating a CORBA adaptation of the requirements of digital video monitoring systems applied to the development of applications with artificial vision functionality. Examples of VISOR-compliant video processing modules are: a motion detector, a people counter, a number plate reader, a face recognizer, and a people tracker. During the period 1990–2000 some interesting video-based surveillance systems of the second generation have been developed within the context of Italian projects. In the Progetto Finalizzato Trasporti II (PFT2) (1993–1996) the University of Genoa developed a vision system for railway level crossing surveillance for supporting a human operator in a remote control room. The system was able to focus the attention of the operator on significant scenes provided by a b/w camera. The surveillance system prototype installed at the Genoa-Rivarolo railway level crossing had the following functionalities: (a) information acquisition from the environment by using various sensors, (b) information processing to identify dangerous situations in monitored areas (level crossing), (c) presentation of alarm situations to the human operator in the control room. In particular, some innovative system modules were implemented: (a) a change-detection module which allows the individuation of changed areas in the current frame with respect to the reference image (background), (b) a localization module which is able to identify and localize on a 2D map all possible objects (e.g., cars, motorcycles, persons) in the monitored area, and (c) an interpretation module able to generate an alarm signal when anomalous situations were detected. Figure 5 shows the man machine interface of the PFT2 visual surveillance system.

**Figure 6.**
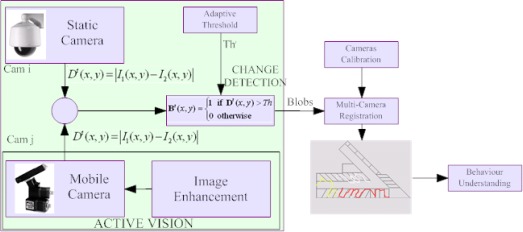
The *PER*^2^ project availed of the dynamic video-surveillance. In particular, static cameras have been supported by PTZ cameras. These, in context of active vision, are able to provide higher details and quality with respect to static ones.

In the last decade, some other video-surveillance systems have been developed in Italy for guarding remote environments in order to detect and prevent dangerous situations. In the context of the research activities financed by the Italian Ministry of University and Scientific Research (MURST), some projects can be analyzed. The Sakbot (Statistical And Knowledge-Based Object deTector) project [32], developed in 2003 by the ImageLab group at the University of Modena and Reggio Emilia, applies computer vision techniques for outdoor (traffic control) and indoor surveillance. Object detection is performed by background suppression and the background is updated statistically. Shadows are detected and removed to increase the accuracy of the object detection process. The *PER*^2^ project, developed in 2003 by a group of four Italian Universities (Genoa, Cagliari, Udine and Polytechnic of Milan), has realized a distributed system for multisensor recognition with augmented perception for ambient security and customization [33]. The aim of this project was to explore innovative methodologies finalized to equip advanced, multi-sensorial surveillance systems with features such as: increased perception and customized communications. These features are indispensable to increase ambient and user security. The augmented degree of perception in the system, achieved by an efficient processing of multi-sensorial data, resulted indispensable to find dangerous situations in complex scenes with a real time behaviour. Moreover, the augmented interaction degree between the system and the customer, obtained by a customized information transmission, also allowed to manage situations of real danger in high level dynamic contexts. In such a project, the adoption of heterogeneous sensors (see Figure 6) allowed to address a wider range of situations thanks to the active control of the fields of view of the surveillance network.

**Figure 7.**
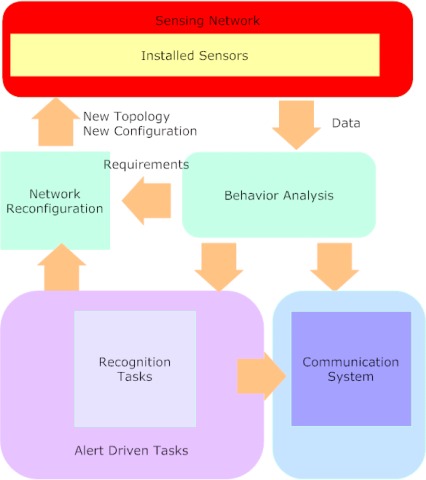
Architecture of the system for Ambient Intelligence.

The studies in the field of active vision, started within the *PER*^2^ project, continued in a follow-up project for the development of Ambient Intelligence techniques for event analysis, sensor reconfiguration and multimodal interfaces. In the period 2007–09, four Italian Universities (Udine, Padova, Pavia and Rome ”La Sapienza”) have contributed to the development of the project ”Ambient Intelligence: Event Analysis, Sensor Reconfiguration and Multimodal Interfaces” [34]. The aim was the study and development of new algorithms and techniques for the design of a network of heterogeneous sensors for automatic monitoring of public environments. The main architecture, presented in Figure 7, manages, data coming from sensors and alarms from selected (of interest) scenarios, as well as alerting operators for ground checking (collecting live data) by mobile devices (carried by guardians). For instance, the automatic detection of an undesired human behaviour can activate the sensors’ network reconfiguration (to improve further detection or recognition) or demand for a guardian to check it for recording a high quality image of the subject’s face. In the period 2006–08, the Rome Public Transportation Company (ATAC) has participated to the CARTAKER (Content Analysis and Retrieval Technologies to Apply Knowledge Extraction to massive Recording) [35] IST European project. The project has developed and assessed multimedia knowledge-based content analysis for automatic situation awareness, diagnosis and decision support in the context of a metroline environment. Recent advances in the research field on visual-based surveillance systems in Italy can be found on the Proceeding of the First Workshop on VIdeoSurveillance projects in ITaly (VISIT 2008).

## 3. Intelligent Visual Sensors

### 3.1. Visual Data Processing at Sensor Level

**Figure 8.**
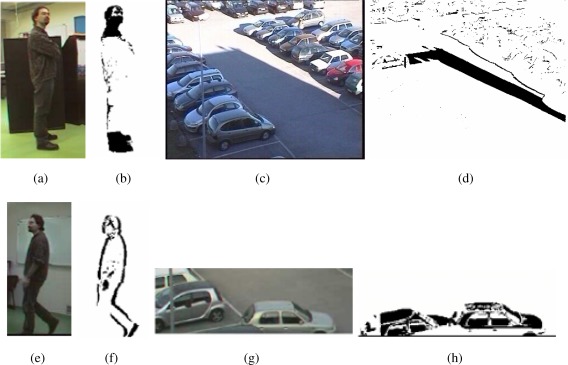
Examples of typical motion detection problems. (a,b)) camouflage, (c,d)) light changes, (e,f) foreground aperture, (g,h) ghosting.

The processing of image sequences acquired by video sensors can be structured in several abstraction layers, ranging from the low-level processing routines in which each image is considered as a group of pixels and basic features need to be extracted (e.g. image edges, moving objects etc.) up to the highest abstraction level in which semantic labels are associated to images and parts of images in order to give a meaningful description of the actions, events and behaviours detected in the monitored scene. Even though the lowest processing level has been widely studied since the beginning of computer vision research, it is still affected by many open problems; actually it is common belief that the major limitations for high-level techniques is the lack of proper low-level algorithms for robust feature extraction. One of the most common low-level problems consists in the detection of moving objects within the scene observed by the sensor, a problem often referred to with the terms change detection, motion detection or background/foreground segmentation. The basic idea is to compare the current frame with the previous ones in order to detect changes, but several problems must be faced, e.g.:

- camouflage effects are caused by moving objects similar in appearance to the background (changes in the scene do not imply changes in the image, e.g., Figure 8 (a) and (b))
- light changes can lead to changes in the images that are not associated to real foreground objects (changes in the image do not imply changes in the scene, e.g., Figure 8 (c) and (d))
- foreground aperture is a problem affecting the detection of moving objects with uniform appearance, so that motion can be detected only on the borders of the object (e.g., Figure 8 (e) and (f))
- ghosting refers to the detection of false objects due to motion of elements initially considered as a part of the background (e.g., Figure 8 (g) and (h))

Change detection algorithms can be roughly classified in two main categories, depending on the elements which are compared in order to detect changes:

- frame-by-frame algorithms
- frame-background algorithms (with reference background image or with background models).

In the first case, moving objects are detected by searching for changes within two or more adjacent frames in the video sequences [36]: if *F_t_*(*x, y*) is a frame at time *t*, the change detection image *D*(*x, y*) is defined as

(1) $$D(x,y)=\;|F_{t}(x,y)-F_{t-1}(x,y)|$$

This technique is typically robust to ghosting effects, but it is generally affected by foreground aperture problems as in Figure 8(f), since two frames both containing the moving object are compared. Frame-background algorithms instead rely on a model representing the background scene without any moving object, and each frame is compared to the model. Background models can be simple images or more complex models containing for example statistical information on the temporal evolution of each background pixel. When using background images, let *B_t_*(*x, y*) be a background frame at time *t*, objects can be detected by image difference:

(2) $$D(x,y)=\;|F_{t}(x,y)-B_{t}(x,y)|$$

or by more complex image comparison techniques, such as Normalized Cross-Correlation [37]. The background image also needs to be constantly updated in order to reflect small changes in the background appearance, for example due to slow light changes in outdoor environments. A typical approach is to apply a running average with exponential forgetting to each pixel value; this is the mean of the measured pixel values by giving more weight to the more recent measures [38]. More complex background models can also be used; it is the case of the popular mixture-of-Gaussian background model proposed by Stauffer and Grimson [39], in which each pixel of the scene is represented by a mixture of several Gaussians, in order to give a proper statistical model for those pixel with multimodal appearance (e.g. flickering screens, waving leaves, etc.).

**Figure 9.**
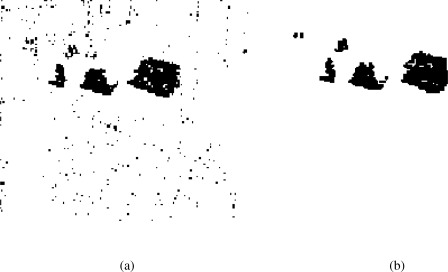
(a) Change detection operation performed on the images in Figure 5, (b) output of the morphological operation.

Moreover, as the output image *D*(*x, y*) of the change detection techniques is a gray-level difference image, thresholding algorithms (i.e., the techniques proposed by Tsai [40], Rosin [41] and Snidaro [42]) must be used to obtain a binary foreground/background image, where changing pixels are set to 1 and background pixels are set to 0. Practically, isolated points represent noise points, while compact regions (blobs) of changed pixels represent possible moving objects in the scene. Noise, artificial illumination, reflections and other effects can create a non uniform difference image, where a single threshold cannot locate all object pixels. In order to reduce the noise and to obtain uniform and compact regions, blob images can be filtered by using mathematical morphology operators such as erosion and dilation [43]. Mathematical morphology describes images as sets and image processing operators as transformations among sets [44]. Figure 9(a) shows the output of the change detection operation performed on the input and background images of Figure 5, respectively. Figure 9(b) shows the output of the morphological operation.

Detected blobs can be further analyzed in order to assign them to predefined object categories. Powerful local features (i.e., SIFT, etc.) computed for each blob have proven to be very successful in object classification such they are distinctive, invariant to image transformations and robust to occlusions. An exhaustive comparison among different descriptors, different interest regions, and different matching approaches can be found in [45].

### 3.2. Automatic Camera Parameter Regulation

**Figure 10.**
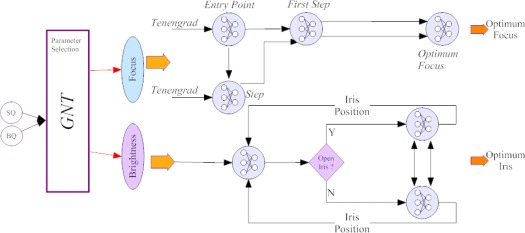
The neural network hierarchy proposed by Micheloni and Foresti in [46].

Video sensors take into consideration only a restricted area around the centre of the image to compute the optimal focus or iris position. Moreover, in context of visual surveillance application the objective is to improve the quality for a human operator. This, not necessarily means that such a quality is optimal for image processing techniques. The method developed in [46] adaptively regulates the acquisition parameters (i.e. focus, iris) by applying quality operators on the object of interest. Hence, the control strategy is based on a hierarchy of neural networks trained on some useful quality functions and on camera parameters (see Figure 10).

Such a solution allows to drive the regulation of the image acquisition parameters on the basis of the target quality. It is interesting to notice how two different hierarchies are involved depending on the desired task. If the object of interest is out of focus, the hierarchy responsible of the focus tuning is activated. Depending on the defocusing degree of the object the systems requires only four (for really out of focus objects) or two (for slightly out of focus objects) steps to bring the object inside and optimal focus range.

**Figure 11.**
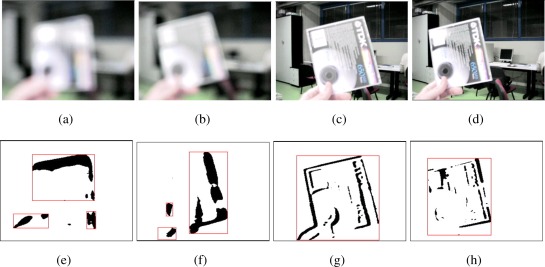
Example of focusing of a moving object. In the first frame (a), the acquisition quality does not allow to accurately detect and recognize the object (e) (smooth contours). After four steps, the quality of the object of interest is much greater (d) and also its detection is improved (h) (sharp contours).

**Figure 12.**
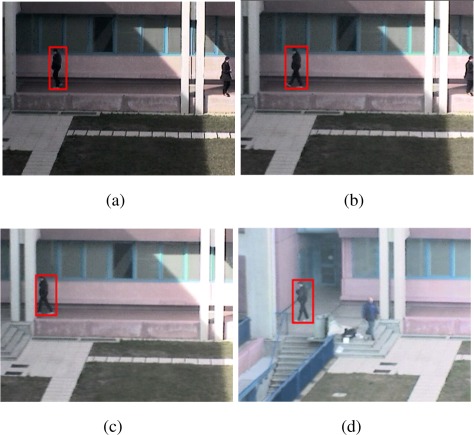
Example of brightness control. As the target walks in a dark area (a), the system requests two consecutive opening of the iris on the basis of the targets brightness only (b) and (c). While the brightness of the object is considered appropriate by the system (d), the remaining of the image is overexposed from a human point of view.

In Figure 11, some results of the strategy proposed in [46] for the focus regulation are presented. It is interesting to notice how in this case, from a starting frame in which the object of interest is really out of focus, by applying the four step strategy it is possible to focus on the object of interest in only four steps. Such a result is even more interesting when applied on tracked features [47] for egomotion compensation [48]. On the other hand, when the strategy requires to adjust the brightness of the target (Figure 12), the proposed solution first decides if the iris must be opened or closed then the amount of motion in the selected direction. This process would not finish as it is not possible to determine an optimal brightness value but it is possible only to determine if the new value is better than the previous one. For such a reason the strategy allows just one step of regulation after which the system can decide if another regulation is necessary or if it is better to adjust the focus.

In [49], the problem of variations in operational conditions has been analyzed. This problem requires long set-up operations and frequent intervention by specialized personnel. An autonomic computing system has been developed to reduce the costs of installation by regulating internal parameters, by introducing self-configuration and self-repair of vision systems.

### 3.3. Sensor Selection

The sensor selection problem is well known in the wireless sensors network domain. In the case where a large number of devices is deployed in vast environments, those devices are generally very inexpensive and with limited battery power. The sensor selection task consists in choosing the subset of sensors that optimizes the trade-off between the utility of the data transmitted while observing a given phenomenon and the cost (e.g. battery power consumed). In this section we will concentrate on video sensors. In addition, we will ignore possible constraints such as power consumption or bandwidth occupancy. We will instead consider only the utility factor of the data transmitted, not its cost. This means defining a way to measure the performance of a camera in performing a certain task. For example, if multiple sensors are monitoring the same scene, redundant data (i.e. positions of the objects) can be fused to improve tracking [50]. The fusion process necessarily has to take into account a quality factor for each sensor, not to have the fused result swayed by unreliable data. Evaluating the performance of a video sensor can be a difficult task, though. It depends on the application and on the type of information that needs to be extracted from the sensors [51]. Until recently, most of the work has concentrated on metrics used to estimate the quality of source images corrupted by noise or compression when the flawless original image is available [50, 52]. Specifically dealing with surveillance applications, the evaluation of the results obtained after the source images have been processed (i.e. to perform change detection [1, 53]) is an important step to consider to assess the performance of a sensor. Segmentation algorithms can be tested off-line on sequences for which a reference segmentation is available [54]. However, for on-line systems, since no reference segmentation is available at run-time, the quality of the results must be estimated in absolute terms. Only recently the problem of estimating segmentation quality without ground truth as been addressed [50, 54, 55]. Since no reference segmentation is used, the measures rely on several comparisons between the pixels of the detected object and those of the background [50]. Other techniques may involve the comparison between two consecutive frames of the color histogram of the object or of its motion vectors [55]. The evaluation of the segmentation can be performed globally on the entire scene or individually for each detected object [55]. The former computes a global quality index for all the blobs detected in the scene. The latter expresses a quality figure for each one of them.

**Figure 13.**
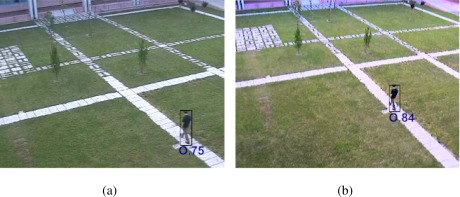
Individual segmentation quality of a moving person detected by two sensors (bounding boxes and segmentation scores are visualized on the source frames). According to the metric proposed in [50], the second sensor (b) provides a better detection as the target and the background are more contrasted.

In Figure 13, an individual segmentation quality was computed according to the metric used in [50] which is based on frame-background difference. The quality is expressed as index ranging from 0 (worst) to 1 (best). In the figure, two sensors are observing the same scene from different view angles. The images produced by the second sensor (b) have more contrast and the walking man can be better discriminated from the background with respect to the first sensor (a). This condition is reflected by the quality indexes shown below the bounding boxes. This quality measure was used in [50] to assess the uncertainty related to the detected object and therefore the performance of the sensor in detecting the object. This information was exploited in the fusion process directed to obtain a robust position estimation and tracking of the target. A feature selection mechanism such as the one presented in [56] can also be used to estimate the performance of the sensor in detecting a given target. The approach is able to select the most discriminative features to separate the target from the background by applying a two-class variance ratio to log likelihood distributions computed from samples of object and background pixels.

### 3.4. Performance Evaluation

In order to complete the analysis of visual sensor technology for advanced surveillance systems it is mandatory to briefly describe evaluation methods that can be used to measure video processing performance. Standard evaluation methods depend heavily on the testing video sequences that can contain different video processing problems such as environmental conditions (e.g., fog, heavy rain, snow, etc.), illumination changes, occlusion, etc. In [57], a new evaluation methodology able to isolate each video processing problem and define quantitative measures to compute the difficulty level of processing a given video has been presented. Specific metric measures have been also presented to evaluate the algorithm performance relatively to the problems of handling weakly contrasted objects and shadows.

## 4. Conclusions

In this paper, a brief historical view of visual-based surveillance systems from the origins to nowadays has been presented together with a short description of the main research projects in Italy on surveillance applications in the last twenty years. The principal technological aspects of advanced visual-based surveillance systems have been analyzed and particular emphasis has been taken on advanced visual sensor networks describing the main activities in this research field. In addition, recent trends in visual sensors technology and recent processing techniques able to increase the quality of acquired data have been described. In particular, advanced visual-based procedures able automatically modify intrinsic and extrinsic camera parameters and automatically select the best subset of sensors in order to monitor a given object moving in the observed environment have been presented.
